# Deep Learning-Assisted Localization of Cystic Lesions and Benign Tumors in the Maxillofacial Region Using Panoramic Radiographs: A Preliminary Feasibility Study

**DOI:** 10.3390/jcm15072784

**Published:** 2026-04-07

**Authors:** Kai-Hua Lien, Sih-Yi Wu, Yun-Ya Yang, Jia-Yu Liu, Yi-Cheng Chen, Ten-Yi Huang, Yu-Wen Tang, Yen-Chu Hsiao, Chung-Bin Wu, Cheng-Chia Yu

**Affiliations:** 1 Department of Stomatology, Division of Oral and Maxillofacial Surgery, Taichung Veterans General Hospital, Taichung 407, Taiwan; k.h.lien@vghtc.gov.tw (K.-H.L.);; 2 School of Dentistry, Chung Shan Medical University, Taichung 402, Taiwan; 3 Department of Electrical Engineering, National Chung Hsing University, Taichung 402, Taiwan; 4 X Intelligence Inc., Hsinchu County 302, Taiwan; 5 Department of Dentistry, Chung Shan Medical University Hospital, Taichung 402, Taiwan; 6 Institute of Oral Sciences, Chung Shan Medical University, Taichung 402, Taiwan

**Keywords:** deep learning, Mask R-CNN, cystic lesions, benign tumors, panoramic radiographs, odontogenic cysts, ameloblastoma, instance segmentation, maxillofacial pathology, artificial intelligence in dentistry

## Abstract

**Background/Objectives:** Automated localization of cystic lesions and benign tumors on panoramic radiographs may support lesion recognition in the maxillofacial region. This preliminary feasibility study aimed to develop and evaluate a deep learning model based on Mask R-CNN for the localization of dentigerous cysts (DCs), radicular cysts (RCs), odontogenic keratocysts (OKCs), and ameloblastomas using panoramic radiographs. **Methods:** A total of 215 panoramic radiographs were retrospectively collected from Taichung Veterans General Hospital (2018–2023). After excluding postoperative, recurrent, or low-quality images, 184 lesions were allocated to the training set and 47 lesions to the testing set. Lesions were annotated based on pathology-confirmed diagnoses. The Mask R-CNN model was trained to localize and classify four lesion types. Model performance was evaluated using precision, sensitivity (recall), and F1 score at an Intersection over Union (IoU) threshold of 0.1. **Results:** In the testing set (*n* = 47), 26 lesions were correctly localized, yielding an overall sensitivity of 55.3% and a precision of 83.9%. The corresponding F1 score was 66.7%. Lesion-specific sensitivities were 40.0% for ameloblastomas, 37.5% for OKCs, 36.8% for RCs, and 93.3% for DCs. **Conclusions:** This study suggests the preliminary feasibility of a deep learning-assisted approach for lesion localization on panoramic radiographs. However, the absence of lesion-free control images and the limited dataset size restrict the generalizability and clinical applicability of the findings. Further validation using larger and more balanced datasets is required.

## 1. Introduction

Artificial intelligence (AI), introduced in 1956, has become integral in various fields, including healthcare, where it plays a crucial role in tasks such as classification, object detection, and image segmentation [1,2,3]. The rise of deep learning, especially convolutional neural networks (CNNs), has revolutionized medical imaging. CNNs automatically ex-tract features from raw data, which allows them to outperform traditional machine learning methods [4]. In CNNs, the convolutional layers perform automated and hierarchical extraction of spatial features, eliminating the need for manual feature engineering. In contrast, traditional machine learning methods such as support vector machines or decision trees rely heavily on manually selected features, which often limits model performance [5]. In dentistry, AI has been applied to areas such as the detection of dental caries, periodontal disease, and periapical lesions, improving diagnostic accuracy and reducing clinician workload [6].

Panoramic radiography remains an essential diagnostic tool in oral and maxillofacial pathology, although identifying early-stage odontogenic cysts and tumors remains challenging. Features such as asymptomatic presentation and early-stage indistinguishability in common cysts, including dentigerous cysts (DCs) and odontogenic keratocysts (OKCs), often delay diagnosis, potentially leading to larger lesions and requiring more extensive surgical interventions [7]. Despite increasing interest in AI-based dental imaging, studies specifically focusing on instance-level localization of multiple odontogenic cystic lesions on panoramic radiographs remain limited.

Mask R-CNN is a robust deep learning model widely used in computer vision for both object detection and instance segmentation [8]. Specifically, Mask R-CNN extends Faster R-CNN by incorporating an additional segmentation branch, enabling simultaneous object detection and pixel-level boundary delineation. Compared with bounding-box-only models such as YOLO or segmentation-only architectures such as U-Net, Mask R-CNN provides instance-level discrimination, which may be advantageous when multiple lesions are present or when precise lesion boundaries are required for further analysis. This characteristic makes it a technically appropriate candidate for exploratory evaluation in the context of odontogenic lesion localization.

However, most existing studies emphasize detection accuracy within broader datasets or focus on classification tasks, and relatively few have examined feasibility in a positive-only, pathology-confirmed dataset with detailed lesion-level annotation. Therefore, the present study aimed to evaluate the preliminary feasibility of applying a Mask R-CNN-based model for the localization of four types of jaw lesions—DCs, radicular cysts (RCs), OKCs, and ameloblastomas—on panoramic radiographs. Model performance was assessed using precision, sensitivity (recall), and F1 score. This study was designed as an exploratory feasibility investigation rather than a validation of clinical diagnostic performance.

## 2. Materials and Methods

### 2.1. Patient Selection and Data Collection

This study included panoramic radiographs of patients diagnosed with cystic lesions or benign tumors. The diagnoses were confirmed by pathological histology at the Department of Oral and Maxillofacial Surgery, Taichung Veterans General Hospital, from 2018 to 2023. A total of 215 panoramic radiographs (231 lesions) were selected from 310 patients with 337 lesions. The lesions comprised 26 ameloblastomas, 24 OKCs, 82 DCs, and 99 RCs. Ameloblastoma cases included both unilocular and multilocular radiographic presentations, without further subtype stratification. Lesion size was not incorporated as a separate analytical variable in this study. Only initial preoperative radiographs were included; detailed inclusion and exclusion criteria are summarized in Table 1. After reviewing the images, 95 were excluded, resulting in a final dataset of 215 panoramic radiographs. For model development and evaluation, lesion-level annotations were used, and each annotated lesion was treated as an individual instance for analysis. The data were randomly divided into training and testing sets.

Due to the retrospective design, data splitting was performed at the image level rather than through pre-specified patient-level stratified randomization. However, all images belonging to the same patient were allocated to the same subset (training or testing), and no patient appeared in both subsets. Therefore, direct cross-subset patient overlap did not occur. As a result, the distribution of lesion types across the two sets was not statistically uniform (Table 2). This imbalance reflects real-world clinical prevalence, especially for less common lesions such as ameloblastomas and OKCs.

### 2.2. Image Annotation

The panoramic radiographs were annotated using the Mask R-CNN model, with labels based on the confirmed pathological diagnoses. Lesions were classified into four categories: DCs, RCs, OKCs, and ameloblastomas.

### 2.3. Image Pre-Processing and Data Augmentation

To improve the quality of the radiographs, pre-processing steps included deblurring, contrast adjustment, and image sharpening. Additionally, a Laplacian operator was used for automatic image classification and sharpening, reducing manual effort [9]. To further enhance the dataset’s diversity, data augmentation techniques [10]—such as adjusting brightness, contrast, rotation, flipping, cropping, and resizing—were applied. Synthetic image generation using Generative Adversarial Networks (GANs) was not implemented in the present study. All images used for model training and evaluation were original clinical radiographs.

### 2.4. Construction of the Deep Learning Algorithm

**Figure 1:**
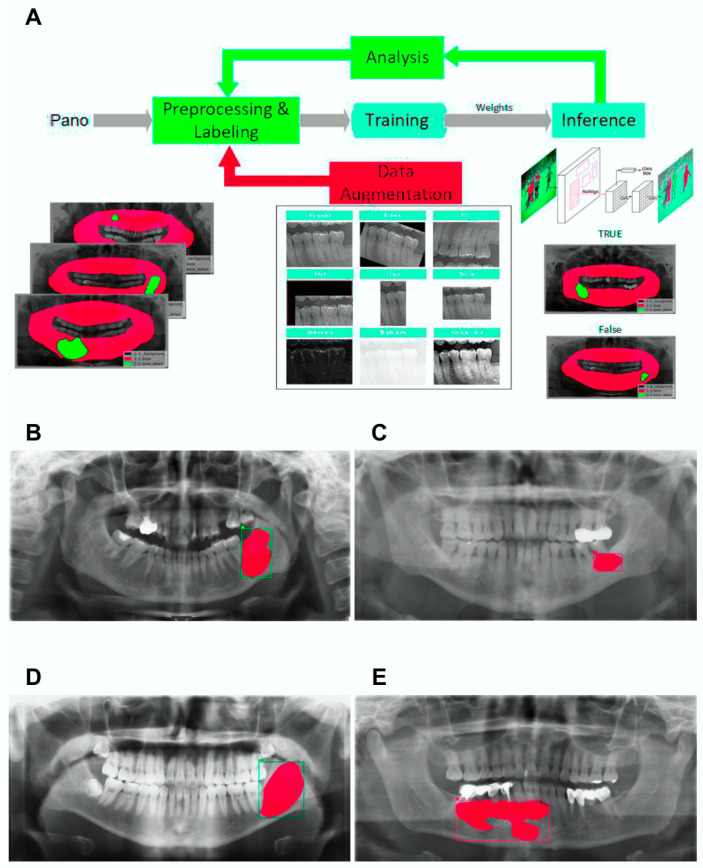
Representative workflow and segmentation outcomes for each lesion type. (**A**) Workflow of the deep learning pipeline using Mask R-CNN. The process includes image preprocessing and labeling, data augmentation, model training, inference, and analysis. Part of panel (**A**) was adapted from He et al. [8]. (**B**) Example of a dentigerous cyst labeled using instance segmentation. (**C**) Example of a radicular cyst. (**D**) Example of an odontogenic keratocyst. (**E**) Example of an ameloblastoma. In subfigures (**B**–**E**), the red shaded areas represent the predicted instance segmentation masks of the lesions, while the colored rectangular boxes indicate the corresponding bounding boxes identified by the model. Each color of the bounding box corresponds to a specific lesion category (e.g., green for DCs, yellow for OKCs). All examples are overlaid with predicted segmentation masks on panoramic radiographs.

The Mask R-CNN architecture was implemented in this study (Figure 1). Given the presence of adjacent teeth and surrounding bony structures that may interfere with lesion detection in panoramic radiographs, an instance segmentation framework was considered appropriate. The model training and inference were implemented based on the SOLOv2 algorithm within the AdelaiDet open-source toolbox (available at https://github.com/aim-uofa/AdelaiDet, accessed on 20 March 2026).

- OS (Operating System): Ubuntu 16.04 (Canonical Ltd., London, UK);
- Programming Language: Python 3.7 (Python Software Foundation, https://www.python.org);
- Packages: PyTorch (version 1.10; available at https://pytorch.org, accessed on 20 March 2026), Pip, TensorFlow (version 2.0; Google LLC, Mountain View, CA, USA), Keras (version 2.3.1; available at https://keras.io, accessed on 20 March 2026);
- GPU (Graphics Processing Unit): NVIDIA GeForce RTX 3090 (NVIDIA Corp., Santa Clara, CA, USA), CUDA (Compute Unified Device Architecture) 10.2, driver 470.256.02, cuDNN 7.6.5;
- Database: MySQL (version 8.0.19; Oracle Corp., Austin, TX, USA);
- Labeling Tool: LabelMe (version 5.0.1; available at https://github.com/wkentaro/labelme, accessed on 15 March 2026).

### 2.5. Mask R-CNN Architecture and Workflow

The study aims to detect and classify odontogenic cysts and tumors from panoramic radiographs. Key objectives are:

- Utilizing Mask R-CNN for image analysis and feature identification.
- Assessing deep learning’s effectiveness in classifying cysts and tumors.
- Speeding up the training process with a GPU and finding optimal parameter combinations.
- Developing models with higher detection performance through extensive data training.

As illustrated in Figure 1, the workflow starts with preprocessing, including deblurring and enhancement. Suitable images are selected and labeled, generating annotation files. These, along with the images, are used for model training. Upon completing training, weight files are created, which are tested and evaluated. If results are unsatisfactory, issues are analyzed and addressed, possibly by augmenting the dataset through various methods, followed by retraining.

### 2.6. Statistics and Performance Evaluation

To evaluate the performance of our deep learning model, we analyzed the sensitivity and model performance across different lesion types. Given the dataset constraints, the following statistical metrics were computed:

- True Positives (TP): 26 lesions correctly localized and correctly classified.
- False Negatives (FN): 21 lesions that were not correctly localized and classified, including undetected and misdiagnosed cases.
- False Positives (FP): 5 predictions localized to incorrect anatomical sites (wrong site or background).

Sensitivity (recall) was calculated as the ratio of true positives to the sum of true positives and false negatives, corresponding to 26 correctly identified lesions out of 47 total lesions in the testing set, yielding a sensitivity of 55.3%.

Precision was calculated as the proportion of correctly localized and classified lesions among all predicted positive localizations. Based on 26 true positives and 5 false positives, the resulting precision was 83.9%.

The F1 score was calculated as the harmonic mean of precision and sensitivity. Using the corresponding values of 83.9% and 55.3%, the F1 score was 66.7%.

This result was derived from the structured testing set and corresponds to the overall evaluation metrics defined in Table 3, which include sensitivity, precision, and F1 score, following standard definitions commonly used in machine learning literature [3].

Since the dataset did not include lesion-free control images, True Negatives (TNs) were not available, and specificity was not calculated.

Further lesion-specific performance details are summarized in Table 4.

To evaluate localization performance, Intersection over Union (IoU) was calculated at the mask level to assess the overlap between predicted and ground-truth masks. Ground truth labels were reviewed and confirmed by two oral and maxillofacial surgeons using clinical notes, panoramic radiographs, computed tomography (CT) scans, and pathology reports.

True positives were defined as predicted masks with an IoU ≥ 0.1 relative to the ground truth and correct lesion classification. Given the relatively indistinct lesion boundaries on panoramic radiographs and the exploratory nature of this feasibility study, an IoU threshold of 0.1 was selected to reflect coarse, clinically relevant localization rather than pixel-level segmentation overlap; we acknowledge that this threshold is conceptually lenient and therefore interpret results at IoU = 0.1 strictly as exploratory feasibility indicators. Performance at a higher threshold (0.3) was also reported for comparison. Based on this criterion, model performance metrics were computed (Table 3).

Mean IoU (mIoU) was calculated as the average IoU across correctly localized lesions. When the IoU threshold was set to 0.1, the model achieved a mean IoU of 0.5258 ± 0.2398. Raising the threshold to 0.3 reduced the mean IoU to 0.4832 ± 0.2524. These comparisons are summarized in Table 5 (*p* = 0.7913). Future studies incorporating negative controls or independent manual validation of AI predictions could provide further insights.

## 3. Results

### 3.1. Model Performance

The Mask R-CNN model was evaluated using the IoU metric for each lesion type across a testing set of 47 panoramic radiographs (Table 5). When the IoU threshold was increased to 0.3, the mean IoU decreased slightly compared with the threshold of 0.1. At an IoU threshold of 0.1, the model achieved a sensitivity of 55.3% and a precision of 83.9%, with a corresponding F1 score of 66.7%. A total of 26 lesions out of 47 were correctly localized and classified (Table 4). The lesion-specific sensitivities for the four lesion types were as follows: 40.0% for ameloblastomas, 37.5% for OKCs, 36.8% for RCs, and 93.3% for DCs.

### 3.2. Analysis of Lesion Types

The model exhibited varying levels of performance across different lesion types (Figure 1). For DCs, the sensitivity was higher, reaching 93.3%. In contrast, other lesion types showed lower sensitivities, suggesting greater difficulty in accurately localizing and classifying these lesions.

### 3.3. Exploratory Analysis of Labeling Strategy

**Figure 2:**
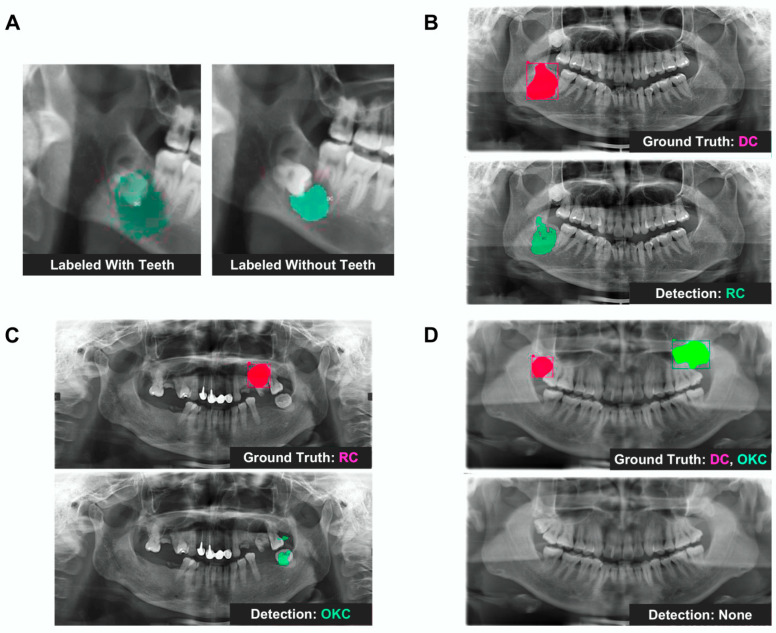
Representative examples illustrating the impact of labeling strategy and model prediction errors. (**A**) Comparison of dentigerous cysts (DCs) labeled with (**left**) and without (**right**) inclusion of associated teeth. In an exploratory experiment conducted during model development, incorporating adjacent teeth into the annotation was associated with an increase in sensitivity from 53.0% to 87.0%. This comparison reflects an earlier experimental configuration and is presented to illustrate the potential influence of labeling strategy. (**B**) Example of misclassification: the lesion was correctly localized but misclassified (DC labeled as RC). (**C**) Positional misidentification: both the lesion location and classification were incorrect. (**D**) Complete detection failure: two lesions (DC and OKC) were labeled in the image, but neither was detected. This image was not part of the dataset used for training or evaluation and is shown solely for illustrative purposes.

In a preliminary exploratory experiment conducted during model development, incorporating the associated teeth into the labeling process for DC cases was associated with an increase in sensitivity from 53% to 87%. This comparison was performed under an earlier experimental configuration and is presented to illustrate the potential impact of labeling strategy (Table 6, Figure 2). The final model results reported in Table 4 reflect the optimized labeling protocol.

### 3.4. Summary of Performance Metrics

Table 3 summarizes the precision, sensitivity, and F1 score for each lesion type. Precision values were numerically higher than sensitivity across lesion types; however, these precision estimates should be interpreted cautiously because lesion-free control images were not included and false-positive behavior could not be evaluated. Ameloblastomas showed lower sensitivity compared with DCs and other lesion types. Overall, the model demonstrated higher sensitivity for DCs, whereas performance for other lesion types remained more limited, indicating areas for further optimization.

## 4. Discussion

The results of this study suggest preliminary feasibility of applying a Mask R-CNN model to localize cystic lesions and benign tumors in panoramic radiographs. The overall sensitivity at an IoU threshold of 0.1 was 55.3%, with a precision of 83.9% and an F1 score of 66.7%. Given the relatively small testing set (*n* = 47), these estimates should be interpreted with caution. As shown in Table 4, model performance varied across different lesion types. However, performance metrics under an IoU threshold of 0.1 should be interpreted as reflecting coarse localization feasibility rather than clinically precise segmentation accuracy.

Identifying odontogenic cysts and tumors in clinical practice often requires significant experience due to ambiguous radiographic features. The limited availability of specialists can lead to delays or misdiagnoses, though final diagnosis is confirmed by pathology. In the past decade, artificial intelligence has significantly advanced in dentistry [4,7,11,12,13,14,15,16]. Among recent studies on odontogenic cysts, Sivari et al. highlighted that deep learning applications in oral and dental health can reduce the workload of professionals by providing more comprehensive, reliable, and accurate image evaluation and disease detection, while also lowering costs and improving access to diagnosis and treatment in underserved areas [6]. Feher et al. [11] conducted a multicenter study using 855 panoramic images for training and 384 for evaluation. Their findings indicated that combining object detection with image segmentation could enhance diagnostic reasoning, achieving an average precision of 0.42 and sensitivity of 0.84 for odontogenic cysts. Notably, their study incorporated an international human control group, consisting of dental professionals from multiple countries, to compare AI-assisted diagnosis with human expert performance.

Unlike their approach, which integrated detection and segmentation separately, our study applied Mask R-CNN as an end-to-end solution for simultaneous detection and localization of lesions. The differences in model architecture and dataset composition may account for variations in sensitivity and precision across studies. Additionally, Feher et al. utilized a larger dataset, which may have contributed to improved generalizability [11].

Yang et al. [7] conducted a study at Yonsei University Dental Hospital, analyzing 1602 lesions from 2010 to 2019. They compared the real-time object detecting deep convolutional neural network You Only Look Once (YOLO)—a deep learning algorithm that can both detect and classify an object at the same time—with oral and maxillofacial surgeons and general practitioners and reported that YOLO achieved high accuracy and diagnostic efficiency. This suggests potential for AI systems to assist general dentists in diagnosing odontogenic cysts and tumors with high accuracy.

The potential ability of AI models to assist in lesion localization may contribute to earlier recognition; however, clinical validation remains necessary before such systems can be applied in real-world diagnostic settings. In our study, an exploratory experiment conducted during model development showed that the sensitivity for DCs increased from 53% to 87% when associated teeth were included in the labeling process (Figure 2, Table 6). This comparison reflects an earlier experimental configuration and is presented to illustrate the potential influence of labeling strategy.

Collaboration between AI researchers and dental professionals is crucial to ensure that deep learning models align with real-world diagnostic requirements. Future studies should also evaluate the clinical usability of AI-assisted diagnostics by incorporating user feedback from radiologists and oral surgeons.

This preliminary study has several limitations. First, the overall dataset size was limited, particularly for rare lesions such as ameloblastomas and OKCs, which may have limited generalizability. Second, the distribution of lesion types between the training and testing sets was statistically imbalanced (Table 2) due to lesion-level allocation and the natural rarity of certain lesions. Although this may introduce potential bias, data augmentation was applied to increase training diversity; however, augmentation does not fully resolve dataset imbalance or the statistical instability associated with small subgroups. Lesion-specific metrics (Table 4) are therefore presented for descriptive, exploratory interpretation. Third, the dataset included only pathologically confirmed positive cases, without any lesion-free images. As a result, specificity and false-positive rates could not be evaluated, which may limit applicability in screening scenarios where distinguishing normal from abnormal is required. Fourth, multiple lesions from the same patient were treated as independent instances, potentially introducing intra-patient correlation. Future studies could consider patient-level partitioning or hierarchical modeling. Fifth, variations in image quality, the presence of adjacent anatomical structures, and postsurgical changes may have affected lesion visibility and contributed to missed or misclassified detections. Sixth, to ensure consistency, radiographs with poor quality, mixed dentition, or postsurgical alterations were excluded, potentially limiting the dataset’s representativeness for broader clinical populations. Seventh, lesion annotations were not independently reviewed by board-certified oral pathologists, which may have affected annotation accuracy and consistency. Lastly, this study utilized only panoramic radiographs. Incorporating multimodal imaging such as CT or magnetic resonance imaging (MRI) in future studies may improve model robustness and clinical utility. Despite these limitations, our findings suggest the feasibility of deep learning in assisting the detection of maxillofacial lesions and highlight the need for further clinical validation.

## 5. Conclusions

This study demonstrates preliminary feasibility in localizing and classifying cystic lesions and benign tumors in panoramic radiographs using a Mask R-CNN-based model. At an IoU threshold of 0.1, the model achieved an overall sensitivity of 55.3%, a precision of 83.9%, and an F1 score of 66.7%. While precision values were numerically high under the study’s positive-only evaluation setting, they should be interpreted cautiously due to the absence of lesion-free controls; sensitivity varied across lesion types, with more limited performance observed for lesions such as ameloblastomas.

Given the limited dataset and the absence of lesion-free controls, the findings should be interpreted as exploratory evidence of localization feasibility rather than validation of diagnostic performance. However, rigorous validation with negative controls and larger, balanced datasets is essential before clinical application can be considered.

Future research should focus on dataset expansion, including the incorporation of lesion-free control images, as well as model optimization and improved annotation strategies to enhance clinical applicability. With further development, AI models like Mask R-CNN may assist in lesion detection in maxillofacial pathology and support clinical decision-making.

## Figures and Tables

**Table 1:** Inclusive Criteria and Exclusive Criteria for Image Selection.

Inclusive Criteria	Exclusive Criteria
Pathological report including dentigerous cyst, radicular cyst, odontogenic cyst, odontogenic keratocyst, ameloblastoma Preoperative panoramic image	Recurrent lesions Postoperative images Complicated recognition of lesions: include dental follicle (uncomplete root formation), multiple primary teeth, and blurry images

**Table 2:** Lesion-Level Distribution in the Training and Testing Sets.

	Ameloblastoma	OKC	DC	RC	Total	*p* Value
*n* (%)	26 (11.3%)	24 (10.4%)	82 (35.5%)	99 (42.9%)	231 (100%)	
Group						0.006 **
Training (*n*/%)	21 (80.8)	16 (66.7)	67 (81.7)	80 (80.8)	184 (79.7)	
Testing (*n*/%)	5 (19.2)	8 (33.3)	15 (18.3)	19 (19.2)	47 (20.3)	
Testing success (*n*/%)	2 (40.0)	3 (37.5)	14 (93.3)	7 (36.8)	26 (55.3)	
Testing failure (*n*/%)	3 (60.0)	5 (62.5)	1 (6.7)	12 (63.2)	21 (44.7)	
Reason for failure (*n* = 21)						0.034 *
Wrong site	0 (0.0)	0 (0.0)	0 (0.0)	5 (41.7)	5 (23.8)	
Not detected	3 (100.0)	2 (40.0)	1 (100.0)	7 (58.3)	13 (61.9)	
Misclassified as another lesion	0 (0.0)	3 (60.0)	0 (0.0)	0 (0.0)	3 (14.3)	

Statistical comparisons were conducted using the chi-square test, Fisher’s exact test, and one-way ANOVA. * *p* < 0.05, ** *p* < 0.01. Percentages in the “Reason for failure” section are calculated based on the number of testing failures for each lesion type. All values are reported at the lesion level rather than the patient level. Abbreviations: DC, dentigerous cyst; OKC, odontogenic keratocyst; RC, radicular cyst.

**Table 3:** Definitions of Performance Metrics Used for Model Evaluation. Definitions adapted from standard classification metrics commonly used in machine learning literature [3].

**Component**	**Definition**
True Positive (TP)	Lesions correctly localized and correctly classified
False Negative (FN)	Lesions not correctly localized or detected with incorrect classification
False Positive (FP)	Predictions localized to incorrect anatomical sites (wrong site or background)
**Metric**	**Definition**
Sensitivity (Recall)	Proportion of correctly identified lesions among all ground-truth lesions
Precision	Proportion of correctly identified lesions among all predicted positive localizations
F1 Score	Harmonic mean of precision and sensitivity

**Table 4:** Classification Results by Lesion Type under an IoU Threshold of 0.1.

Training Epoch: 3500	IoU Threshold: 0.1				
	mAP 0.071				
	Lesion Type				Total
	Ameloblastoma	OKC	RC	DC	
Training (n)	21	16	80	67	184
Testing (n)	5	8	19	15	47
Sensitivity (%)	40.0	37.5	36.8	93.3	55.3
Precision (%)	100.0	100.0	58.3	100.0	83.9
F1 score (%)	57.1	54.5	45.2	96.6	66.7

Classification performance of the Mask R-CNN model is presented for each lesion type using an IoU threshold of 0.1. Precision and F1 score reflect the model’s ability to balance true positive and false positive predictions, while mAP summarizes overall model performance across lesion types based on the precision–recall relationship. Performance metrics are defined in Table 3. Abbreviations: DC, dentigerous cyst; IoU, intersection over union; mAP, mean average precision; OKC, odontogenic keratocyst; RC, radicular cyst.

**Table 5:** Segmentation Results at IoU Thresholds of 0.1 and 0.3 for Overall Model Performance.

Training Epoch: 3500	IoU Threshold: 0.1	IoU Threshold: 0.3
	mAP 0.071	mAP 0.071
Training (n)	184	184
Testing (n)	47	47
Sensitivity (%)	55.3	34.0
Precision (%)	83.9	94.1
F1 score (%)	66.7	50.0

Segmentation results for all lesion types are presented under two IoU thresholds: 0.1 and 0.3. The IoU metric quantifies the overlap between predicted and ground-truth lesion boundaries, with higher values indicating better segmentation. These comparisons are summarized in Table 5 (*p* = 0.791). Performance metrics including precision, sensitivity, and F1 score are defined in Table 3. Abbreviations: IoU, intersection over union; mAP, mean average precision.

**Table 6:** Impact of Teeth Inclusion on Segmentation Performance of Dentigerous Cysts.

Annotation Type	Training (n)	Testing (n)	Sensitivity (%)
Label with teeth	67	15	87.0
Label without teeth	67	15	53.0

Segmentation performance for DCs is compared between annotations with and without inclusion of adjacent teeth. In an exploratory experiment conducted during model development, incorporating associated teeth into the labeling was associated with higher sensitivity (87.0% vs. 53.0%). This comparison reflects an earlier experimental configuration and is presented to illustrate the potential influence of labeling strategy. The results correspond to the left and right panels in Figure 2A. Training epoch: 3500; IoU threshold: 0.1. Abbreviations: DC, dentigerous cyst; IoU, intersection over union.

## Data Availability

All data are included within the article.

## Abbreviations

The following abbreviations are used in this manuscript:

AI	Artificial Intelligence
CNNs	Convolutional Neural Networks
CT	Computed Tomography
DCs	Dentigerous Cysts
FN	False Negative
FP	False Positive
GPU	Graphics Processing Unit
IRB	Institutional Review Board
IoU	Intersection over Union
mAP	Mean Average Precision
OKCs	Odontogenic Keratocysts
RCs	Radicular Cysts
TP	True Positive
YOLO	You Only Look Once

## References

[1] Buchanan B. (2005). A (Very) Brief History of Artificial Intelligence. AI Mag..

[2] Thurzo A., Urbanová W., Novák B., Czako L., Siebert T., Stano P., Mareková S., Fountoulaki G., Kosnáčová H., Varga I. (2022). Where Is the Artificial Intelligence Applied in Dentistry? Systematic Review and Literature Analysis. Healthcare.

[3] Sadr S., Mohammad-Rahimi H., Motamedian S.R., Zahedrozegar S., Motie P., Vinayahalingam S., Dianat O., Nosrat A. (2023). Deep Learning for Detection of Periapical Radiolucent Lesions: A Systematic Review and Meta-analysis of Diagnostic Test Accuracy. J. Endod..

[4] Bayrakdar I.S., Orhan K., Çelik Ö., Bilgir E., Sağlam H., Kaplan F.A., Görür S.A., Odabaş A., Aslan A.F., Różyło-Kalinowska I. (2022). A U-Net Approach to Apical Lesion Segmentation on Panoramic Radiographs. Biomed. Res. Int..

[5] LeCun Y., Bengio Y., Hinton G. (2015). Deep learning. Nature.

[6] Sivari E., Senirkentli G.B., Bostanci E., Guzel M.S., Acici K., Asuroglu T. (2023). Deep Learning in Diagnosis of Dental Anomalies and Diseases: A Systematic Review. Diagnostics.

[7] Yang H., Jo E., Kim H.J., Cha I.-H., Jung Y.-S., Nam W., Kim J.-Y., Kim J.-K., Kim Y.H., Oh T.G. (2020). Deep Learning for Automated Detection of Cyst and Tumors of the Jaw in Panoramic Radiographs. J. Clin. Med..

[8] He K., Gkioxari G., Dollar P., Girshick R. (2020). Mask R-CNN. IEEE Trans. Pattern Anal. Mach. Intell..

[9] Acharya T., Ray A.K. (2005). Image Processing: Principles and Applications.

[10] Shorten C., Khoshgoftaar T.M. (2019). A survey on Image Data Augmentation for Deep Learning. J. Big Data.

[11] Feher B., Kuchler U., Schwendicke F., Schneider L., de Oro J.E.C.G., Xi T., Vinayahalingam S., Hsu T.-M.H., Brinz J., Chaurasia A. (2022). Emulating Clinical Diagnostic Reasoning for Jaw Cysts with Machine Learning. Diagnostics.

[12] Çelik B., Savaştaer E.F., Kaya H.I., Çelik M.E. (2023). The role of deep learning for periapical lesion detection on panoramic radiographs. Dentomaxillofac. Radiol..

[13] Kumar V.S., Kumar P.R., Yadalam P.K., Anegundi R.V., Shrivastava D., Alfurhud A.A., Almaktoom I.T., Alftaikhah S.A.A., Alsharari A.H.L., Srivastava K.C. (2023). Machine learning in the detection of dental cyst, tumor, and abscess lesions. BMC Oral Health.

[14] Poedjiastoeti W., Suebnukarn S. (2018). Application of Convolutional Neural Network in the Diagnosis of Jaw Tumors. Healthc. Inform. Res..

[15] Song I.-S., Shin H.-K., Kang J.-H., Kim J.-E., Huh K.-H., Yi W.-J., Lee S.-S., Heo M.-S. (2022). Deep learning-based apical lesion segmentation from panoramic radiographs. Imaging Sci. Dent..

[16] Ver Berne J., Saadi S.B., Politis C., Jacobs R. (2023). A deep learning approach for radiological detection and classification of radicular cysts and periapical granulomas. J. Dent..

